# Spectral Estimation Model Construction of Heavy Metals in Mining Reclamation Areas

**DOI:** 10.3390/ijerph13070640

**Published:** 2016-06-28

**Authors:** Jihong Dong, Wenting Dai, Jiren Xu, Songnian Li

**Affiliations:** 1 School of Environment Science and Spatial Informatics, China University of Mining & Technology, Xuzhou 221116, China; daiwentingcumt@126.com (W.D.); snli@ryerson.ca (S.L.); 2 Jiangsu Key Laboratory of Resources and Environmental Information Engineering, China University of Mining & Technology, Xuzhou 221116, China; 3 School of Geography, University of Leeds, Leeds LS2 9JT, UK; jirenxu@126.com; 4 Department of Civil Engineering, Ryerson University, Toronto, ON M5B 2K3, Canada

**Keywords:** mining area, reclamation soil, heavy metal, spectrum, estimation model

## Abstract

The study reported here examined, as the research subject, surface soils in the Liuxin mining area of Xuzhou, and explored the heavy metal content and spectral data by establishing quantitative models with Multivariable Linear Regression (MLR), Generalized Regression Neural Network (GRNN) and Sequential Minimal Optimization for Support Vector Machine (SMO-SVM) methods. The study results are as follows: (1) the estimations of the spectral inversion models established based on MLR, GRNN and SMO-SVM are satisfactory, and the MLR model provides the worst estimation, with *R*^2^ of more than 0.46. This result suggests that the stress sensitive bands of heavy metal pollution contain enough effective spectral information; (2) the GRNN model can simulate the data from small samples more effectively than the MLR model, and the *R*^2^ between the contents of the five heavy metals estimated by the GRNN model and the measured values are approximately 0.7; (3) the stability and accuracy of the spectral estimation using the SMO-SVM model are obviously better than that of the GRNN and MLR models. Among all five types of heavy metals, the estimation for cadmium (Cd) is the best when using the SMO-SVM model, and its *R*^2^ value reaches 0.8628; (4) using the optimal model to invert the Cd content in wheat that are planted on mine reclamation soil, the *R*^2^ and *RMSE* between the measured and the estimated values are 0.6683 and 0.0489, respectively. This result suggests that the method using the SMO-SVM model to estimate the contents of heavy metals in wheat samples is feasible.

## 1. Introduction

Land reclamation in mining areas is a priority for agricultural production in China [1]. However, the characteristics of the reclamation process and materials (i.e., filling the depressions with coal gangue), and the complexity of the reclamation environment often result in heavy metal pollution in the soil, which may directly or indirectly threaten human health by direct contact, through the food chain or in other ways [2,3,4,5,6]. Due to the complex spatial heterogeneity of soil, the chemical analysis methods that have been traditionally used to detect the heavy metal content are found to be laborious, inefficient in terms of time required, and not suitable for large-scale monitoring [7,8,9]. Therefore, how to monitor soil heavy metal pollution quickly and accurately has become an important research topic in the field of mine reclamation.

Reflectance spectral characteristics are basic soil characteristics. Near-infrared reflectance spectroscopy was first used to estimate the heavy metal content in lake sediments to prove its feasibility in investigating heavy metal content [10]. Using a remote sensing spectral analysis method to estimate the content of heavy metals in soil can overcome the shortcomings of traditional sampling methods and monitor the heavy metal pollution in soil dynamically and quickly on a large-scale. Many studies have already been done on the spectral characteristics of soil. The early focus was mainly on soil classification and the factors influencing the soil spectrum [11,12,13,14,15,16,17,18,19]. With continuous research development, researchers began to combine mathematical analysis methods with spectral characteristics, focusing on the quantitative investigation of the physical and chemical properties of soils [20,21,22,23,24,25,26,27]. Although heavy metals have limited effect on the soil spectral curve because they are not the main components of soil, a suitable spectral inversion model can be established and effectively used to estimate the content of heavy metals in soil based on the information-rich spectral characteristics of soil. Currently, many inversion models have been developed, which can be mainly divided into two types—statistical analysis models and machine learning models. The statistical analysis models have the characteristics of simple structure, few parameters and a relatively easy process. For example, multiple linear regression can describe the linear connection between several independent variables and a dependent variable; and the partial least squares method, first proposed in 1983 [28,29], is a type of deformation of multiple linear regression models focusing on the characteristics of principal component analysis, canonical correlation analysis and linear regression analysis methods in the modeling process [30,31]. The machine learning models mainly refer to the artificial neural network (ANN) and support vector machine (SVM) methods. The artificial neural network is a system that imitates the structure and function of nerve cells in human brains [32]. It has been widely used in many fields and achieved satisfactory results due to its ability to perform highly nonlinear mapping [33]. The support vector machine was first proposed in 1995 and its main advantages are that it can solve small sample, nonlinear and high-dimensional pattern recognition problems very well, and can be applied to other machine learning problems, e.g., function fitting [34,35].

Taking the artificial reclamation areas (coal gangue reclamation area and fly-ash reclamation area) of the Liuxin mining area in Xuzhou, China as the case study, this paper establishes quantitative models to simulate the connection between heavy metal contents in mine reclamation soil and characteristic spectral remote sensing parameters, compares the estimation results of different models, and selects the optimal model for the estimation of Cd content in wheat planted in the reclaimed mine soil. The paper further proposes a quick and efficient method that is suitable for large-range monitoring of the heavy metal pollution in mine reclamation soils, as well as the technical support required for the regulation of heavy metal pollution and food security in mining areas.

## 2. Study Area and Data Collection

### 2.1. Study Area

**Figure 1:**
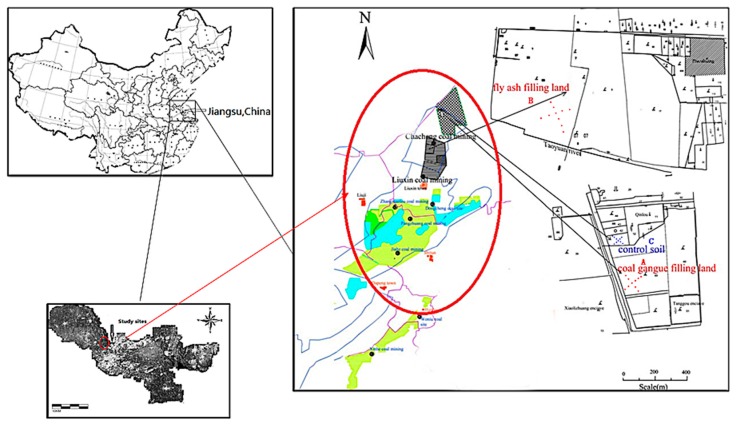
Location and maps of the study area.

The study area is in the Liuxin national reclamation demonstration area of Tongshan County, which is located 20 km northwest of Xuzhou, China. The area has a temperate, humid to semi-humid continental monsoon climate with an average annual precipitation of 800–930 mm, 56% of which falls in July and August. The annual average air temperature is 14 °C with lowest temperature ranging from −9 °C to −13 °C and highest temperature from 36 °C to 39 °C. The climate conditions are suitable for the growth of crops. There are five large coal mines and two large power plants, Chacheng and Huarun, in the demonstration area, and also large areas of subsided lands affected by many years of mining (Figure 1). The coal mining subsidence was used for agriculture production after filling the reclamation area with mainly coal gangue and fly ash. The collapsed area in the coal gangue reclamation area (Zone A), backfilled in 1998, was directly filled with coal gangue of different block sizes and then covered with 40–45 cm soil for subsequent planting. The collapsed area in the fly-ash reclamation area (Zone B), backfilled in 1999, was directly filled with power plant fly ash and then covered with 40–50 cm soil for subsequent planting. In addition, an area with a soil depth greater than 1 m was used as the experimental control soil (Zone C). The control area has the same climatic conditions, tillage methods, planting crops and the influence groundwater as those of the reclamation areas. The cultivation system of the study area follows the wheat and rice rotation mode.

### 2.2. Spectral Data Collection

Field sampling was completed in March 2012 and April 2012. The surface soil samples in the coal gangue reclamation area, fly-ash reclamation area and control area were designated and collected according to the plum blossom stationing method. Ten sampling points were determined in each area, and a total of 30 soil samples were collected.

#### 2.2.1. Heavy Metal Content Measurement

A traditional chemical detection method was used to measure the contents of heavy metals in the soil samples. The soil sample pretreatment included air drying, grinding, mesh screening, digesting and constant volume adjustment. After pretreatment, the heavy metal contents in the soil samples were detected by an Inductively Coupled Plasma-Mass Spectrometry (ICP-MS, Agilent, Palo Alto, CA, USA).

#### 2.2.2. Outdoor Spectral Data Collection

The outdoor spectral data of the soil samples were collected from 11:00 a.m. to 2:00 p.m. using an ASD FieldSpec 3 Spectrometer (ASD Inc., Boulder, CO, USA). A standard white reflection plate was used to calibrate the instrument before collecting each soil sample spectrum. The sampling points were identified with GPS to precisely fix the positions, and 10 spectral curves were collected for each soil sample. The average of the 10 spectral curves for each soil sample was used as the actual reflectance spectrum of the soil samples [36].

#### 2.2.3. Indoor Spectral Data Collection

The soil samples were stored in a darkroom for indoor spectral data collection. The spectrometer was preheated to stability, and the standard white reflection plate was used for instrument calibration. The soil samples were placed on a plain black velvet, the surfaces of the soil samples were flattened before recording the spectral data in dishes. Finally, 10 spectral curves were collected for each soil sample. The arithmetic average of the 10 spectral curves for each soil sample was used as the actual reflectance spectrum of the soil sample.

### 2.3. Data Analysis

#### 2.3.1. Heavy Metal Contents in the Soil Samples

The heavy metal contents in the soil samples from the three areas detected using the traditional chemical detection methods are shown in Table 1. The heavy metal contents in the coal gangue reclamation area are relatively close to the values in the fly-ash reclamation area, and the heavy metal contents in both reclamation areas are obviously higher than those of the control area. Therefore, taking the spectral characteristics and the sampling point quantity into account, the soil samples from the coal gangue and fly-ash reclamation areas are both regarded as the mining area reclamation soil in this research.

#### 2.3.2. Spectral Data of the Soil Samples

**Figure 2:**
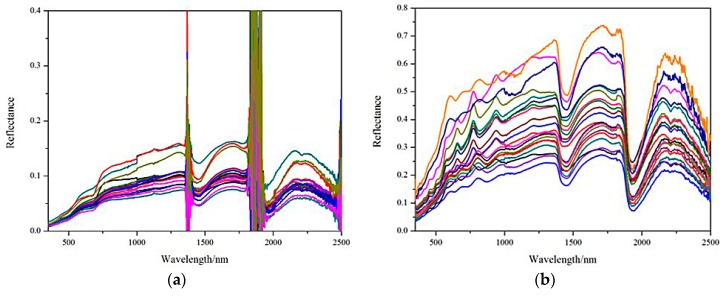
Original outdoor (**a**) and indoor (**b**) spectra of mine reclamation soils.

The original outdoor and indoor spectral curves of the soil samples in the mining area are shown in Figure 2. There are two obvious reflection peaks in the outdoor spectral curve at 1400 nm and 1900 nm, which are influenced by the light intensity, air suspended particles, wind speed, gas and polarization interference of the surrounding targets during the process of the outdoor spectral data collection. In addition, missing or overlapping information in the soils’ typical reflection peaks and absorption valley bands is the most serious problem of the spectral data collected outdoors in this study. It is generally considered that 600 nm and 815 nm are typically the reflection peak and the second reflection peak of the soil organic matter, respectively, while the weak absorption peak of Fe^2+^ and Fe^3+^ is near 900 nm and the absorption peak near 1000 nm is the characteristic spectral band of iron hydroxides in soil. The outdoor spectral data largely lose these spectral features. The indoor spectral curve is relatively smooth, and there are no abnormal reflection peaks. Because of the large differences in soil conditions between the control area and the reclamation area, this paper selected the indoor spectral data of the two mine reclamation soils for the characteristics extraction and the spectral estimation model construction.

## 3. Research Methods

### 3.1. Multivariable Linear Regression

Multivariable linear regression, MLR, is used to predict the independent variable Y by establishing the regression equation model with the optimal combination of multiple dependent variables *X*. The MLR model is a classical statistical analysis method based on the least squares method. The basic form is:

(1) $$Y=\beta_{0}+\beta_{1}X_{1}+\cdots \beta_{j}X_{j}+\cdots \beta_{n}X_{n}+\varepsilon$$

where *Y* represents the characteristics to be analyzed; $X_{j}$ represents the *j*th independent variable; $\beta_{j}$ represents the regression coefficient corresponding to the *j*th independent variable; and ε represents a random error of the regression equation, subject to the normal distribution with a mean of zero, where E(ε)=0; and n represents the number of independent variables used for the modeling.

First, the overall parameters $\beta =\left(\beta_{0},\beta_{1},\cdots ,\beta_{n}\right)$ are estimated based on the least square method. The estimated quantity of *β* is denoted as $\;B=\left(b_{0},b_{1},\cdots ,b_{n}\right)$; therefore, the estimated quantity of *Y* is:

(2) $$\hat{Y}=XB$$

Since the difference between the estimated quantity $\hat{Y}$ and the original vector *Y* must be minimized, the least squares method is used again to calculate the overall parameters of the least squares estimated quantity:

(3) $$B_{LS}={\left(X^{\prime }X\right)}^{-1}X^{\prime }Y$$

Thus, the least squares estimated quantity of *Y* is obtained:

(4) $$\hat{Y}=X{\left(X^{\prime }X\right)}^{-1}X^{\prime }Y$$

In Equation (4), $\text{ X}=X_{1},X_{2},X_{3},\cdots ,X_{n}$, where the rank of *X* is n, does not have a complete correlation. 

### 3.2. Generalized Regression Neural Network

The generalized regression neural network, GRNN, is usually used for function approximation. The entire network consists of four layers: input layer, pattern layer, summation layer and output layer.

The network input is $X={\left[x_{1},x_{2},\cdots ,x_{n}\right]}^{T}$, and its output is $\;Y={\left[y_{1},y_{2},\cdots ,y_{k}\right]}^{T}$. The number of neurons in the input layer is equal to the dimension *m* of the input vector in the model sample. Each neuron can be viewed as a simple distribution unit, which can directly transmit the input variable into the hidden layer. The number of neurons in the model layer is equal to the number *n* of the model sample, and each neuron in this layer corresponds to a model sample. The transfer function of the *i*th neuron in the model layer is:

(5) $$P_{i}=\text{exp}\left[-{\left(X-X_{i}\right)}^{T}\left(X-X_{i}\right)/2\sigma^{2}\right]$$

In Equation (5), X represents the input variable of the network; $X_{i}$ represents the corresponding training sample of the *i*th neuron; and σ represents smoothing parameter. The *i*th output neuron is the exponential form of the Euclidean distance square between the input variable X and the corresponding training sample $X_{i}$:

(6) $$D_{i}^{2}={\left(X-X_{i}\right)}^{T}\left(X-X_{i}\right)$$

The summation layer contains two types of neurons. One type performs the arithmetic summation of all the output of the model layer, and the connection weight between each neuron and this neuron in the model layer is 1. The transfer function is:

(7) $$S_{A}=\sum_{i=1}^{n}P_{i}$$

The other type performs a weighted summation of all the output of the pattern layer. The connection weight between the *i*th neuron in pattern layer and the *j*th neuron in the summation layer is the *j*th element $y_{ij}$ of the *i*th output sample $\;Y_{i}$. The transfer function of the neurons in the summation layer is:

(8) $$S_{Nj}=\sum_{i=1}^{n}y_{ij}P_{i}\;j=1,2,\cdots ,k$$

The number of neurons in the output layer is equal to the dimension *k* of the output vector in the model sample. The output of each neuron is obtained by dividing the two different types of neuron outputs in the summation layer:

(9) $$y_{i}=\frac{S_{Nj}}{S_{A}}\text{ j}=1,2,\cdots ,k$$

Therefore, it can be determined that the structure and weight of the GRNN are fully determined after the selection of the model sample. As a result, the GRNN is more convenient than the other neural networks.

### 3.3. Sequential Minimal Optimization for Support Vector Machines

Sequential minimal optimization for support vector machines, SMO-SVM, is a statistical learning method based on the statistical theory of the VC dimension (for Vapnik–Chervonenkis dimension) theory and the structural risk minimization principle. The basic principle of a standard support vector machine is mapping the n-dimensional sample vector from the original $R^{n}$ space into the feature space *F* through the nonlinear mapping ψ and constructing the optimal linear decision function using the structural risk minimization principle:

(10) $$f\left(x\right)=\omega^{T}\cdot \psi \left(x\right)+b$$

In Equation (10), $x\in R^{n}$, ω∈F, and *b* represents the threshold. For the standard support vector machine, the optimization problem is:

(11) $$\min J=\frac{1}{2}\omega^{T}\omega +C\sum_{i=1}^{S}\left(\xi_{i}+\xi_{i}^{*}\right)$$

The expressions $\left\{ \begin{array}{l} y_{i}-\omega^{T}\psi \left(x_{i}\right)-b\leq \varepsilon +\xi_{i}\\ \omega^{T}\psi \left(x_{i}\right)+b-y_{i}\leq \varepsilon +\xi_{i}\\ \xi_{i},\xi_{i}^{*}\geq 0 \end{array}\right.$ define the formula; *C* represents the penalty coefficient; $\xi_{i},\xi_{i}^{*}$ represent the slack variable; and ε represents an insensitive parameter.

A Lagrange function is established as:

(12) $$L_{p}=\frac{1}{2}{\left\|W\right\|}^{2}-\sum_{i=1}^{n}a_{i}\left[\omega^{T}\psi \left(x_{i}\right)+b+\xi_{i}-y_{i}\right]$$

In Equation (12), $a_{i}$ represents the Lagrange multiplier factor.

The specific steps of the sequential minimal algorithm are as follows:

(1) Select two updated elements $\;a_{1}$ and $\;a_{2}$, order $\;F=w\cdot x_{i}-y_{i}$, and calculate the upper bound H and lower bound L;
(2) Update the element $\;a_{2}$:
  (13) $$a_{2}^{new}=a_{2}^{old}-y_{2}\left(F_{1}^{old}-F_{2}^{new}\right)/k$$
  In Equation (13), $k=k\left(x_{1},x_{1}\right)+k\left(x_{2},x_{2}\right)-2k\left(x_{1},x_{2}\right)$.
(3) If $\Delta a_{2}$ is less than the threshold, the update fails; otherwise, the update element *a*_1_ is as follows:
  (14) $$a_{1}^{new}=a_{1}^{old}+s\left(a_{2}^{old}-a_{2}^{new}\right)$$
  In Equation (14), $s=y_{2}y_{1}$.
(4) Update all the $F_{i}$:
  (15) $$F_{i}^{new}=F_{i}^{old}+\left(a_{1}^{new}-a_{1}^{old}\right)y_{1}k\left(x_{1},x_{i}\right)+\left(a_{2}^{new}-a_{2}^{old}\right)y_{2}k\left(x_{2},x_{i}\right)$$
(5) Calculate the error *E* of the function output and the target classification:
  (16) $$E=\sum_{i=0}^{1}a_{i}y_{i}F_{i}+\sum_{i=0}^{1}\varepsilon_{i}$$
(6) The algorithm ends, if *E* is less than the threshold; otherwise, repeat Equations (1)–(5).

## 4. Spectral Estimation Modelling of Heavy Metals in Mine Reclamation Areas

### 4.1. Stress Sensitive Band Selection of Heavy Metal Pollution

This study carried out a correlation analysis between the content of heavy metal in soils and the spectral reflectance obtained after performing a first-order differential transformation, envelope elimination and inverse logarithmic transformation. Eight maximum correlated bands were selected as pollution stress sensitive bands for the heavy metals Cd, Cr, Cu, Pb and Zn. 

Compared with the first-order differential transformation and envelope elimination, the correlation coefficient between the Cd content in the mine reclamation soil and the spectrum obtained after performing the inverse logarithmic transformation was significantly reduced, and no significant correlation to the selection of feature bands was found. The selection results of the five heavy metals’ stress sensitive spectral bands are shown in Table 2.

### 4.2. Establishment of the MLR Estimation Model

According to the results of the stress sensitive band selection, the spectral reflectance values obtained through the spectral transformation of the sensitive band were defined as a set of independent variables $\;X_{1},X_{2},X_{3},\cdots ,X_{n}$, and the heavy metal content in the soil of the mining area was treated as dependent variable *Y* to establish the model.

The stress sensitive bands with a significant correlation were selected as related factors. The data for 6 sample points (12 soil samples, accounting for 60% of the total samples) from the fly-ash and coal gangue reclamation areas were used as the training data, and the remaining eight soil samples (accounting for 40% of the total samples) were used as the testing data. The regression coefficients of the MLR model were calculated, and the stability and forecast accuracy of the model was predicted. The MLR equations for the contents of the five heavy metals are as follows:

(17) $$Y_{Cd}=-47.02-54.30X_{1}-293.99X_{2}+49.61X_{3}-186.84X_{4}\;-1.73X_{5}-67.72X_{6}-0.51X_{7}-34.74X_{8}$$

(18) $$Y_{Cr}=-257.11+90.75X_{1}+85.64X_{2}-110.08X_{3}-2222.20X_{4}+216.62X_{5}+820.02X_{6}+129.64X_{7}+29.49X_{8}$$

(19) $$Y_{Cu}=307.60-378.73X_{1}-345.84X_{2}+30336.18X_{3}+248.18X_{4}-255.29X_{5}+169.65X_{6}+1480.98X_{7}-1908.58X_{8}$$

(20) $$Y_{Pb}=-2499.12+494.63X_{1}+31.73X_{2}-8094.11X_{3}+25556.99X_{4}+1812.74X_{5}+186.18X_{6}-164.84X_{7}+19.03X_{8}$$

(21) $$Y_{Zn}=430.46-21.75X_{1}-619.75X_{2}+23648.34X_{3}-162.99X_{4}\;-12334.67X_{5}+432.00X_{6}+2615.55X_{7}+2631.48X_{8}$$

This paper evaluated the stability of the regression model with the coefficient of determination (*R*^2^) and the accuracy with the root mean square error using the following formulas, respectively:

(22) $$R^{2}=1-\frac{\sum^{\text{​}}{\left(y_{m}-y_{p}\right)}^{2}/\left(N_{c}-k-1\right)}{\sum^{\text{​}}{\left(y_{m}-\bar{y}\right)}^{2}/\left(N_{p}-1\right)}$$

(23) $$RMSE=\sqrt{\sum^{\text{​}}{\left(Y_{m}-Y_{p}\right)}^{2}/N}$$

In Equations (22) and (23), $Y_{m}$ and $Y_{p}$ represent the measured and predicted values of the heavy metal content, respectively; $\bar{Y}$ represents the average of the measured values of the heavy metal contents; $N_{c}$, $N_{p}$ and *N* represent the number of modeling samples, the number of forecast modeling samples and the number of total samples, respectively; and *k* represents the number of the independent variables in the model.

*R^2^* indicates the stability of the model. The closer the value is to 1, the more stable the model is. *RMSE* indicates the accuracy of the model. The smaller the value, the higher the accuracy of the model. The results for estimating the heavy metal contents in the soil samples of the mining area using the MLR model are shown in Table 3.

As shown in Table 3, the MLR model was used to estimate the heavy metal contents in the soil of the mining area. Among them, the Cr estimation result is the best, and the value of its *R*^2^ reaches 0.5976. The estimated values of Cu and Cd are the second best, with *R*^2^ values more than 0.5. The estimated values of Pb and Zn are the worst, with their *R^2^* values reaching 0.4851 and 0.4687, respectively.

**Figure 3:**
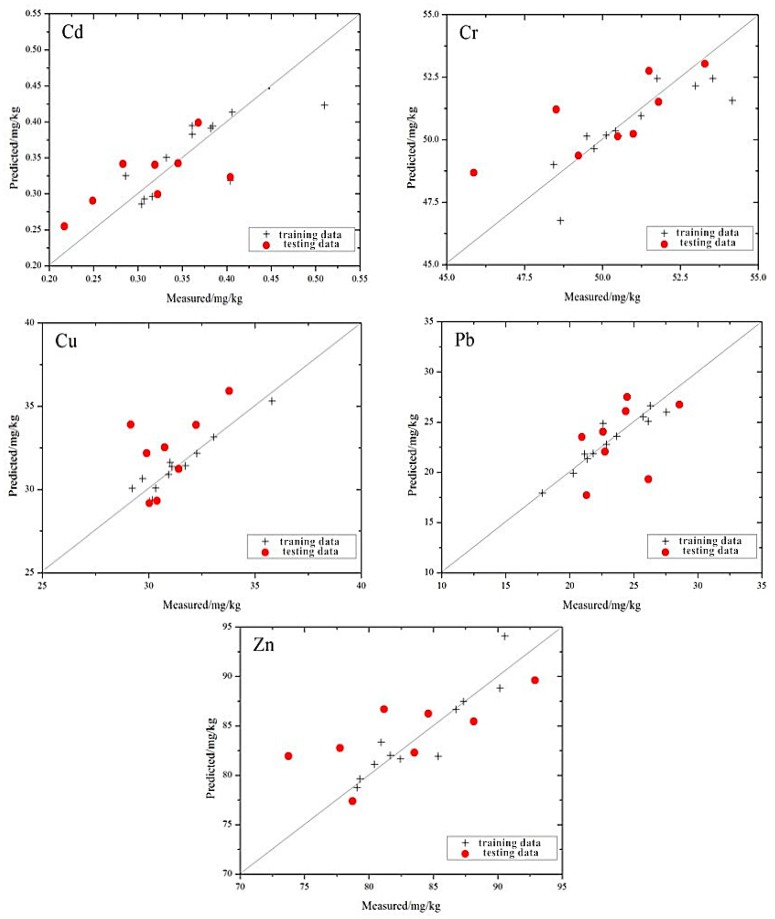
Individual points of heavy metal concentrations of the mine reclamation soils predicted by the MLR model plotted against the measured values.

The distribution of the measured values and the estimated values based on the MLR model is shown in Figure 3. Compared to the prediction efforts of a single element, the distribution of the five types of heavy metal elements is closer to the 1:1 line. Cd, Cr and Zn all have different degrees to which the maximum or minimum values deviate from the 1:1 line. Because the differences between these extreme values and the majority of the sample are great, there are insufficient training samples to support the modeling in the corresponding multi-dimensional space. As a result, the generalization ability of the model is weak, and the model performs unsatisfactorily when the model’s value is a maximum or minimum. 

Among the five heavy metals, the distribution of Cr is the closest to the 1:1 line, and the trend is more consistent with the 1:1 line. Relative to Cr, the distributions of Cd and Cu appear to be loose in the vicinity of the 1:1 line, and the overall trend is consistent with the 1:1 line. The distribution of Pb and Zn show that the estimation of those two heavy metals are not very impressive using the model. These results indicate that the best prediction ability of the model is for Cr, followed by Cu and Cd, while the worst is for Pb and Zn.

### 4.3. Establishment of the GRNN Estimation Model

A smoothing factor σ has a significant effect on the ability for the forecasting and generalization of the GRNN. Therefore, the emphasis rests on improving the accuracy of the network forecasting by selecting and using a suitable smoothing factor as the parameter in this network. The experiment adopted a cross validation approach to conduct the parameter optimization of smoothing factor σ. The steps of the parameter optimization are given as follows:

(1) Set an initializing smoothing factor.
(2) Divide the modeling sample into four equal parts, with the first part as the modeling sample and the rest for establishing the GRNN.
(3) Use the network model established in step (2) to forecast the modeling sample and calculate the *RMSE* in this situation.
(4) Use the second, third and fourth parts to repeat steps (2) and (3), and calculate the average of the *RMSE* for the smoothing factor.
(5) Progressively increase and change the value of the smoothing factor in the proper order; repeat step (2–4); compare the *RMSE* of the network when the smoothing factor takes different values; and take the value of the smoothing factor when the *RMSE* has minimum value as the final value of the smoothing factor of the GRNN.

According to the selection results of the sensitive bands, the spectral reflectance values obtained through the spectral transformation of the sensitive bands were defined as the independent variables $\;X_{1},X_{2},X_{3},\cdots ,X_{n}$, and the heavy metal content in the soil of the mining area is treated as the dependent variable Y to establish the model.

The stress sensitive bands with a significant correlation were selected as the related factors. The data of six sample points (12 soil samples, accounting for 60% of the total samples) from the fly-ash and coal gangue filling sites were considered as the training data, and the remaining eight soil samples (accounting for 40% of the total samples) were considered as the testing data.

**Figure 4:**
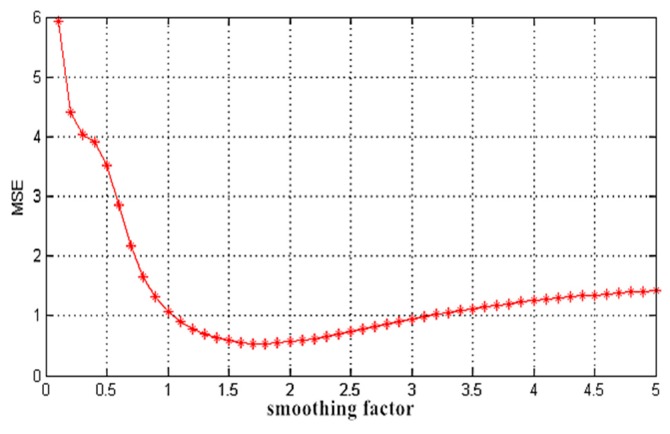
Parameter optimization of the smoothing factor.

The parameter optimization of the smoothing factor σ for Cr in the GRNN is shown in Figure 4. When σ is 1.7, the generalization ability of the network is the best and thus the parameter σ participates in the subsequent process of the regression forecast. The optimization of the other parameters used the same method.

The results using the GRNN model for estimating the five types of heavy metal contents in the reclamation soil of the mining area are shown in Table 4.

As shown in Table 4, the GRNN model can successfully estimate the heavy metal contents of the mine reclamation soils. Among them, the estimated values of Cr and Cd are the best, and their *R^2^* values reach 0.7932 and 0.7843, respectively. The estimated values of Pb and Cu are the second best, with *R*^2^ values greater than 0.7. The estimated value of Zn is the worst, but the *R*^2^ value reaches 0.6990.

**Figure 5:**
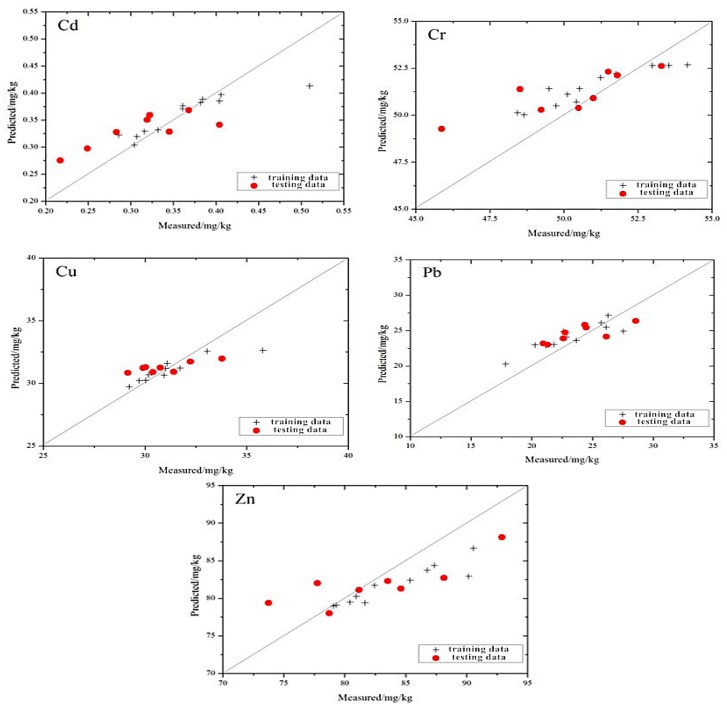
Individual points of heavy metal concentrations of the mine reclamation soils predicted by the GRNN model plotted against the measured values.

The distribution of the measured and the estimated values based on the GRNN model is shown in Figure 5, which shows that the position relationship between the scatter point distribution of the five heavy metals and the 1:1 line is similar to the scatter point distribution of the MLR model. However, the scatter point distribution of the GRNN model is closer to the 1:1 line.

### 4.4. Establishment of SMO-SVM Estimation Model

This experiment selected the Radial Basis Function (RBF) as the kernel function of SMO-SVM. RBF has the characteristics of a good classification effect and easily adjustable parameters. The computational formula is as follows:

(24) $$K\left(x_{i},x_{j}\right)=\exp\left(-\left\|x_{i}-x_{j}\right\|/2\sigma^{2}\right)$$

On selecting the SVM parameters when adopting RBF, the internationally commonly-used method confines the penalty parameter *C* and the kernel function parameter *g* to a certain range of values, uses cross validation to obtain the optimum parameters *C* and *g*, which have the highest accuracy of modeling and validation, and selects the two parameters to participle in the subsequent SVM model establishment. Due to the problem of two-parameter selection, there may be a situation in which there are multiple-unit combinations of *C* and *g* that correspond to the highest accuracy of the validation. Therefore, the strategy of selecting the combination of *C* and *g* can reach the highest validation accuracy when the parameter *C* is minimized. If *g* corresponding to the minimum *C* has several groups, the first *g* that was searched for as the optimal parameter is selected.

The stress sensitive bands with a significant correlation were selected as the related factors. The data of six sample points (12 soil samples, accounting for 60% of the total samples) from the fly-ash and coal gangue filling sites were used as the training data, and the remaining eight soil samples (accounting for 40% of the total samples) were used as the testing data.

**Figure 6:**
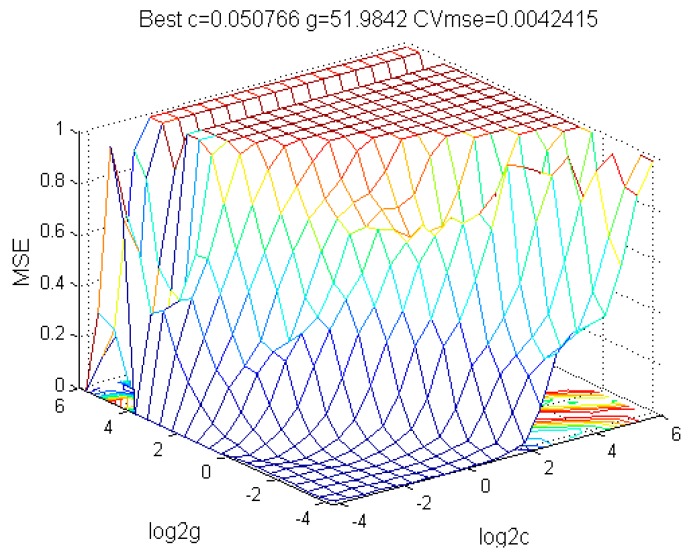
Parameter optimization of *C* and *g*.

The map of parameter optimization of the SVM network’s parameters *C* and *g* for Cr is shown in Figure 6. When C=0.050766 and  g=51.9842, the generalization ability of the support vector machines is the best and thus the parameter combination participates in the subsequent process of the regression forecast. The optimization methods for the rest of the elements are the same.

The results of using the SMO-SVM model for estimating the five types of heavy metal contents in the reclamation soil of the mining area are shown in Table 5.

As shown in Table 5, the SMO-SVM model can successfully estimate the heavy metal contents of the mining area. Among them, the estimated values of Cr and Cd are the best, and their values of *R**^2^*** reach 0.8628 and 0.8532, respectively. The estimated values of Pb and Cu are the second best, with *R*^2^ values greater than 0.79. The estimated values of Zn are the worst, but the value of *R*^2^ reaches 0.7653.

**Figure 7:**
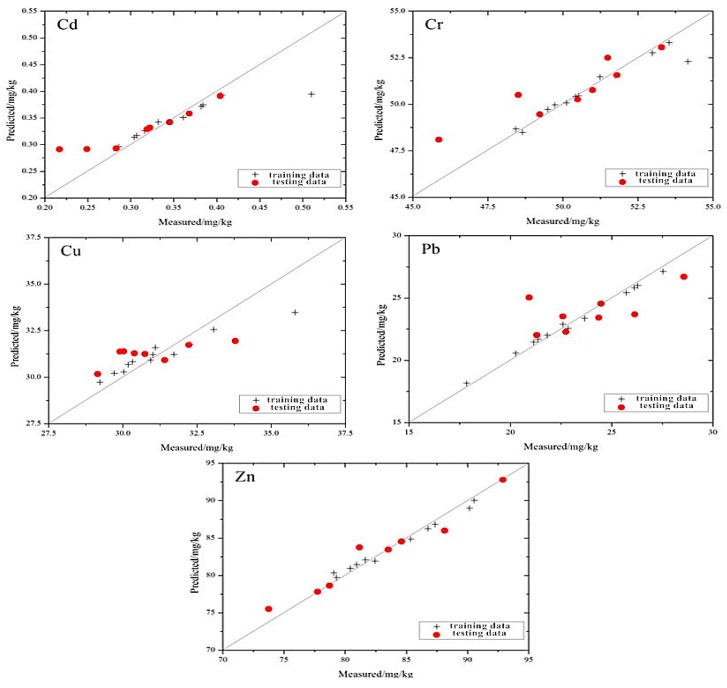
Individual points of heavy metal concentrations in the mine reclamation soils predicted by the SMO-SVM model plotted against the measured values.

The distribution of the measured and the estimated values based on the SMO-SVM model is shown in Figure 7, which shows that the position relationship between the scatter point distribution of the five elements and the 1:1 line is similar to the MLR and GRNN models. However, the scatter point distribution of the SMO-SVM model is closer to the 1:1 line.

### 4.5. Comparison of the Estimation Results of Heavy Metals Based on Different Models

All of the spectral estimation results of heavy metals in the mine reclamation soils based on the three models are shown in Table 6. The SMO-SVM model provides the best estimation of the heavy metal content in the mine reclamation soils, and the stability and accuracy of its estimation results are higher than those of the GRNN and MLR models. Among the five heavy metals, the estimation for Cr using the SMO-SVM model is the best, and its *R*^2^ value reaches 0.8628. The estimation for Cd is second best, and its *R*^2^ reaches 0.8532. The estimation for Zn is the worst, but its *R*^2^ reaches 0.7988. The results show that the estimations of the Cd and Cr contents in the soil samples are relatively good.

## 5. Demonstration of the Optimization Model Used in Estimating the Content of Cd in Wheat

Mine reclamation, especially mine soil reclamation, has been given high priority in agricultural production in China. The heavy metal elements in mine reclamation soil can enter the human body through food crops, therefore, it is significant to study the heavy metal content in crops planted on mine reclamation soils and to evaluate their food safety implications. To demonstrate the feasibility of spectral estimation for the heavy metal contents in crops using the SMO-SVM method, Cd in the wheat planted on mine reclamation soils was selected as the study case.

### 5.1. Determination of the Content of Cd in Wheat

Wheat samples that were planted on the soil sample points were collected at the same time as the collection of the soil samples. The stalk of wheat was collected as the research sample, and 10 wheat samples were collected on each of the three areas. The contents of Cd in the wheat samples are shown in Table 7 according to a traditional chemical analysis method.

### 5.2. Selection of Stress Sensitive Bands of Cd Pollution in Wheat

**Figure 8:**
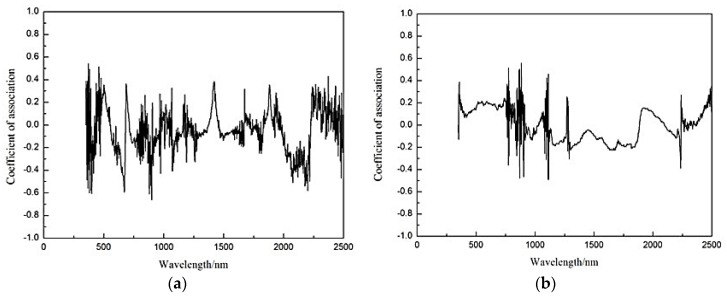
Correlation coefficients of the Cd concentration in the wheat planted on mine reclamation soils with transformed spectra. The spectral reflectance were obtained by performing the first-order differential transformation (**a**) and envelope elimination (**b**) on the corresponding 350–2500 nm wavelength range in the spectral data of the samples.

Because the effect of the spectral inverse logarithmic transformation is relatively unsatisfactory, the original spectral reflectance of the wheat samples was pre-processed by the first-order differential transformation and envelope elimination methods. Correlation analysis was carried out between the content of heavy metal Cd in the wheat samples and the spectral reflectance obtained by performing the first-order differential transformation and envelope elimination on the corresponding 350–2500 nm wavelength range in the spectral data of the samples. The curve of the correlation coefficient varying with wavelength is shown in Figure 8. Figure 8a shows the fluctuation of the relativity between the content of Cd and the spectral reflectance of the wheat after performing the first-order differential transformation varying with wavelength. It presents strong negative correlations at 380, 390, 400, 670, 880, 890, 900 and 2200 nm. All of their values are larger than the maximum correlation coefficient obtained by performing the envelope elimination in Figure 8b. Therefore, the aforementioned eight wavelengths were selected as the stress sensitive bands for Cd pollution in wheat.

### 5.3. Spectral Estimation of the Cd Content in the Wheat Planted in the Reclamation Soil of Mining Areas

**Figure 9:**
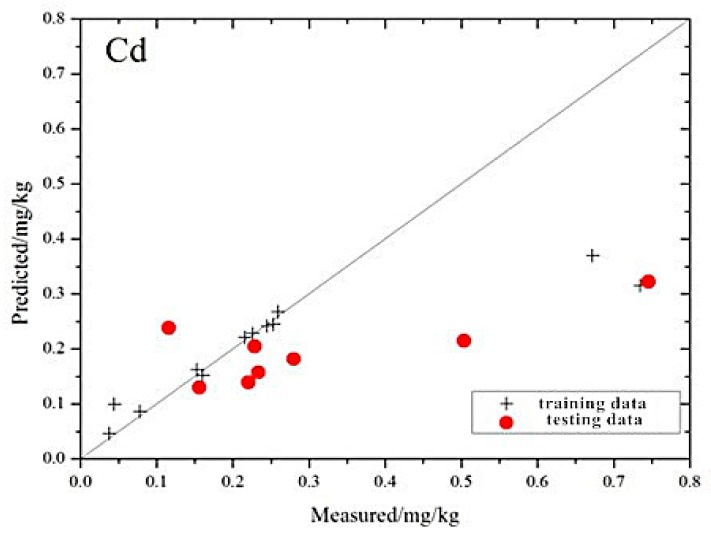
Individual points of the Cd concentration in the wheat planted on mine reclamation soils predicted by the SMO-SVM model against the measured values.

The stress sensitive bands of Cd pollution in wheat were selected as the related factors. The data of six sample points (12 soil samples) from the coal gangue and fly-ash reclamation areas were used as the training data, and the remaining eight samples were used as the testing data. The model was established by using the SMO-SVM method. When the penalty parameter C and the kernel function parameter g are 0.1768 and 2.8284, respectively, through cross validation, the corresponding *RMSE* reaches the minimum value of 0.0446. In this case, the generalization of the support vector machines is the best and thus the parameter combination participates in the subsequent process of the regression forecast. The result is shown in Figure 9.

Using the regression model based on the SMO-SVM method to estimate the Cd’s content in the wheat that were planted in the reclamation soils of the mining area, the correlation coefficient *R^2^* and root mean square error *RMSE* between the measured and the estimated values were found to be 0.6683 and 0.0489, respectively, suggesting that it is feasible to use spectral data to estimate the heavy metal content in the wheat planted in the reclamation soils of mining areas. However, the estimation compared to the soil is relatively unsatisfactory, which may attribute to the fact that the spectral reflectance are affected by multiple components in crop samples, i.e., moisture, chlorophyll, protein and lignin. Further study is needed to develop a more scientific and reasonable method for the spectral estimation of heavy metals in crops.

## 6. Conclusions

In this research, the MLR, GRNN and SMO-SVM models were established to estimate the heavy metal contents in the reclamation soil of mining areas, and the optimal model was found and used to demonstrate its use in estimating Cd in the wheat planted on mine reclamation soils. The spectral inversion models for heavy metals in soil established based on the MLR, GRNN and SMO-SVM perform satisfactory to a certain degree. The *R*^2^ values of the MLR model, which provides the worst estimates, remain above 0.46. This result suggests that the stress sensitive bands of the heavy metal pollution selected by the treatment method of the spectral data and correlation analysis contain sufficient effective spectral information. The established model based on the GRNN has an advantage for modelling small samples in this study. Compared with the MLR model, the GRNN model estimations are superior for the five types of heavy metals in the reclamation soils of mining areas. The stability and accuracy of the SMO-SVM model are the best, compared to those of the GRNN and MLR models. Among the five types of heavy metals, the estimations of Cd and Cr are the best using the SMO-SVM model. Using the optimal SMO-SVM model to estimate the Cd content in the wheat planted in the reclamation soils of mining areas, the *R*^2^ and *RMSE* values between the measured and estimated values are relatively good, suggesting that the method is feasible.

## Figures and Tables

**Table 1:** Heavy metal concentrations of soil samples in the three different areas (mg/kg).

Areas	Sampling Points	Cd	Cr	Cu	Pb	Zn
Coal gangue reclamation area	1	0.384	51.756	29.712	21.162	80.938
	2	0.510	49.496	30.030	22.595	79.296
	3	0.361	54.168	32.268	27.540	82.429
	4	0.404	51.238	35.799	26.125	98.611
	5	0.382	53.544	31.013	26.286	81.651
	6	0.361	52.985	30.178	22.858	79.077
	7	0.322	51.800	31.414	24.476	78.714
	8	0.319	53.292	32.230	28.563	88.128
	9	0.283	48.518	29.153	20.935	73.746
	10	0.368	51.495	30.748	22.595	84.612
Fly-ash reclamation area	1	0.332	50.521	33.060	21.816	90.538
	2	0.304	50.118	30.326	17.847	87.332
	3	0.316	49.734	30.939	25.728	85.356
	4	0.406	48.650	31.093	20.270	90.164
	5	0.286	48.433	29.224	21.370	86.750
	6	0.307	50.419	31.719	23.664	80.423
	7	0.249	49.228	30.391	21.310	77.741
	8	0.404	50.992	33.791	26.145	81.173
	9	0.217	50.491	29.910	24.364	83.532
	10	0.345	45.866	30.033	22.746	92.878
Control area	1	0.098	27.164	11.066	13.030	36.460
	2	0.079	27.173	9.518	11.085	33.156
	3	0.065	30.223	10.822	11.417	73.868
	4	0.104	26.949	10.381	11.071	35.289
	5	0.137	33.536	16.440	11.125	45.329
	6	0.209	30.271	15.919	12.609	43.733
	7	0.193	30.918	14.065	14.595	40.906
	8	0.133	28.210	13.794	12.093	35.852
	9	0.190	27.893	12.224	11.674	34.292
	10	0.112	29.380	12.775	13.031	36.126

**Table 2:** Stress sensitive spectral bands of the five pollution heavy metals (nm).

Heavy Metals	Stress Sensitive Spectral Bands							
Cd	960	1140	1700	1820	2250	2380	2450	2470
Cr	570	670	970	1020	1680	1740	2060	2410
Cu	660	960	1090	1730	1770	1810	2240	2420
Pb	780	960	1090	1280	1680	2160	2380	2480
Zn	490	660	1090	1730	1770	1810	2310	2410

**Table 3:** The estimation results of the MLR model.

Elements	Cd	Cr	Cu	Pb	Zn
*R*^2^	0.5683	0.5976	0.5025	0.4851	0.4687
*RMSE*	0.0438	1.02	1.191	1.997	4.439

**Table 4:** The estimation results of the GRNN model.

Elements	Cd	Cr	Cu	Pb	Zn
*R*^2^	0.7843	0.7932	0.7163	0.7360	0.6990
*RMSE*	0.0310	0.9455	0.8991	1.43	3.341

**Table 5:** The estimation results of the SMO-SVM model.

Elements	Cd	Cr	Cu	Pb	Zn
*R*^2^	0.8628	0.8532	0.7988	0.7901	0.7653
*RMSE*	0.0134	0.7968	0.7570	1.275	2.95

**Table 6:** Estimation results of the heavy metals in the reclamation soils of the mining area based on the three different models.

Elements	Methods	*R*^2^	*RMSE*
Cd	MLR	0.5683	0.0438
	GRNN	0.7843	0.031
	SMO-SVM	0.8628	0.0134
Cr	MLR	0.5976	1.02
	GRNN	0.7932	0.9455
	SMO-SVM	0.8532	0.7968
Cu	MLR	0.5025	1.191
	GRNN	0.7163	0.8991
	SMO-SVM	0.7988	0.757
Pb	MLR	0.4851	1.997
	GRNN	0.736	1.43
	SMO-SVM	0.7901	1.275
Zn	MLR	0.4687	4.439
	GRNN	0.699	3.341
	SMO-SVM	0.7653	2.95

**Table 7:** Cd concentration of the wheat in three different areas (mg/kg).

Sample Points	Coal Gangue Reclamation Area	Fly Ash Reclamation Area	Control Area
1	0.245076	0.160367	0.032794
2	0.225840	0.078045	0.034339
3	0.215431	0.152975	0.103025
4	0.253332	0.037446	0.067843
5	0.259086	0.044392	0.030881
6	0.672122	0.115781	0.083343
7	0.503266	0.233244	0.132664
8	0.734710	0.155536	0.039946
9	0.745570	0.220029	0.096775
10	0.228416	0.280000	0.268284
